# Surface-plasmon-enhanced strain-wave-induced optical diffraction changes from a segmented grating

**DOI:** 10.1016/j.pacs.2023.100497

**Published:** 2023-04-29

**Authors:** Thomas J. van den Hooven, Paul C.M. Planken

**Affiliations:** Advanced Research Center for Nanolithography (ARCNL), Science Park 106, 1098 XG Amsterdam, The Netherlands; Van der Waals-Zeeman Institute, University of Amsterdam, Science Park 904, 1098 XH Amsterdam, The Netherlands

**Keywords:** Ultrafast, Photoacoustics, Surface plasmon polaritons, Segmented grating, Diffraction, Nanostructures

## Abstract

We report on surface-plasmon-polariton-enhanced (SPP-enhanced), strain-wave-induced reflection and diffraction changes on a Au-covered, segmented grating. The segmented grating has a 6020 nm period, and its lines are segmented into 7 periods of a 430 nm period grating, which allows the excitation of SPPs. This grating has three SPP resonances at different optical wavelengths, for the same incident angle. Pump-pulse-induced strain waves are probed by measuring reflection and diffraction of a tunable probe pulse in a wavelength range that includes all three SPP resonances. Surface Acoustic Waves (SAWs) and Longitudinal Waves (LWs) are identified. When probing close to SPP resonances, the reflection changes from SAWs and LWs are strongly enhanced by factors of 23 and 36, respectively, compared with reflection changes observed when probing at off-resonance wavelengths. The relative SAW- and LW-induced diffraction changes are larger by additional factors of up to 3.3 and 2.6, respectively, compared to the reflection changes.

## 1 Introduction

Wafer alignment is a crucial step in the semiconductor device manufacturing process. An important method to align wafers is to measure the phase difference between, for example, the minus and plus first-order diffracted beams of a so-called alignment grating, which is a phase grating etched into the silicon wafer. By measuring changes in this phase difference when the wafer with the grating is displaced along the grating vector, the position of the grating can be determined with (sub-)nanometre accuracy [1].

However, in semiconductor device manufacturing, many layers are deposited onto the wafer, also covering these alignment gratings. Unfortunately, some of these layers can be (partially) opaque to light. In that case, determining the position of the alignment grating can become very challenging. Previously, we have shown that such gratings, buried underneath opaque layers, can be detected using laser-induced strain waves [2], [3]. In these experiments, a pump pulse excites a strain wave near the surface, which travels downward through the stack of layers. The strain wave reflects off the buried grating and, due to the spatially periodic topography of the grating, becomes a strain wave ‘copy’ of the grating. The returning grating-like strain wave deforms the surface and/or changes the refractive index, forming a grating from which a delayed probe pulse can diffract. Although this is a promising technique to detect alignment gratings buried underneath opaque layers, the diffracted signal strength is currently too low for practical applications.

To increase signal strength, we recently showed that the optical detection of strain waves on metallic gratings can be enhanced by probing near a Surface Plasmon Polariton (SPP) resonance [4]. SPPs are EM-waves, travelling along, and bound to, a metal/dielectric interface, coupled to plasma oscillations of the free electrons near the surface of the metal. To excite SPPs, both energy and wavevector conservation is required. For a given incident wavelength this is only achieved for a certain grating period and incident angle. The amplitude and central wavelength of the SPP resonance are changed by the material density changes and by deformations of the grating shape, induced by strain waves. This leads to relatively large variations in the amount of absorbed and reflected light, thus enhancing the optical signal strength.

In the present study, we extend this work to so-called segmented gratings. These gratings, often used in the semiconductor manufacturing industry, are segmented in the sense that the grating lines (ridges) are subdivided into multiple shorter period lines and spaces (valleys). This is typically done to increase the diffraction efficiency of specific orders [5], [6].

Here, the segmented grating consists of an Au-covered, 6020 nm period alignment grating, where each grating line is segmented into seven lines forming a 430 nm period grating that can sustain SPPs. This is mathematically equivalent to amplitude modulation of a high-spatial-frequency grating by a low-spatial-frequency grating, which leads to sidebands in the spatial frequency domain. As a result, white light spectroscopy shows that the segmented grating has not one, but *three* plasmonic resonances for a fixed angle of incidence. A tunable probe pulse is used to probe the segmented grating in the wavelength range from 600 to 705 nm, which includes the three plasmonic resonances at 611, 650, and 695 nm. In the experiment, we not only measure the reflection of the probe pulse from the sample as function of the delay between pump and probe pulse, but also the plus and minus first-order of the probe light diffracted off the long-period alignment grating as a function of pump–probe delay.

Similarly to what was found in earlier experiments on *non*-segmented (plasmonic) gratings [4], we observe several types of strain waves, such as longitudinal waves (LWs) and surface acoustic waves (SAWs). We observe strong enhancements, both in the strain-wave-induced reflection changes and in the strain-wave-induced diffraction changes when tuning the probe wavelength around all three plasmonic resonances. In reflection, the enhancement is a factor of 23 to 36, depending on the type of strain wave, when probing with wavelengths close to the SPP resonance wavelength compared with probing with off-resonance wavelengths. Diffraction measurements show a maximum relative change that is a factor of 3.3 larger for SAWs, compared to the reflection measurements. Surprisingly, we find that the measured SAW period increases with increasing probe wavelength. Furthermore, we observe that the sign of the SAW-induced diffraction, changes around certain probe wavelengths. Finally, we observe that the photo-acoustic signal associated with the longitudinal waves change sign when probing near the SPP resonances. This is caused by strain-wave-induced changes in the position of the SPP resonance. Our results prove that SPPs can be used to enhance photo-acoustic signals on non-continuous, segmented gratings.

**Fig. 1:**
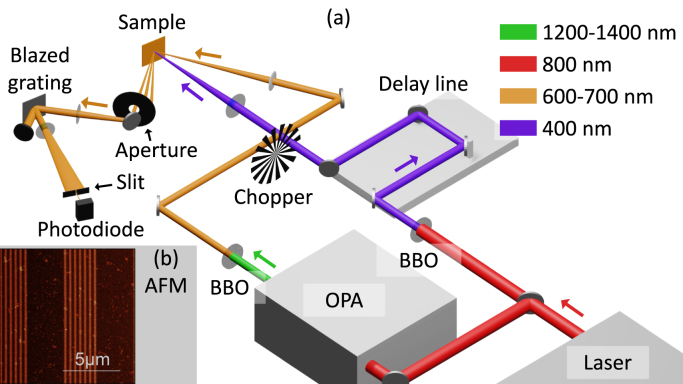
(a) A schematic view of the experimental setup with the used wavelengths, indicated by the differently coloured beams, OPA: Optical Parametric Amplifier, BBO: β-barium borate crystal. (b) AFM-micrograph of the Au-covered segmented grating used in the experiments. (For interpretation of the references to colour in this figure legend, the reader is referred to the web version of this article.)

The structure of this paper is as follows. In Section 2, the experimental setup and the segmented grating sample are briefly described. The results of white light spectroscopy of the segmented grating are presented in Section 3. In Section 4, an analysis of the white light spectroscopy results using a simple model for SPPs is presented. The results of the photo-acoustic experiments are presented and discussed in Sections 5, 6, respectively.

## 2 Experimental setup

A schematic view of the experimental setup is shown in Fig. 1(a). The sample is illuminated by a 400 nm, 50 fs pump pulse at an incident angle of about 5° with respect to the surface normal, which does not excite SPPs. A time-delayed probe pulse, with a central wavelength tunable between 600 and 705 nm, is incident at an angle of 27° with respect to the surface normal and excites SPPs at wavelengths of approximately 611, 650, and 695 nm. After illuminating the sample, an aperture selects one diffraction order and the light is spectrally filtered to reduce the bandwidth to about 2 nm full-width half maximum (FWHM). For more details, we refer to Appendix A.

The segmented grating was made by NanoPHAB BV (Eindhoven, the Netherlands) using e-beam lithography, and is etched into a layer of 200 nm SiO_2_ on a Si substrate. Using thermal evaporation, a layer of Au with an thickness of 172(3) nm is deposited onto the grating. This thickness was determined via a photo-acoustic measurement (see Appendix C for more details). The amplitude of the grating is about 31 nm and the duty cycle of the 430 nm grating is 44 %. An atomic-force microscopy (AFM) image of the Au-covered grating is shown in Fig. 1(b).

## 3 White light spectroscopy

**Fig. 2:**
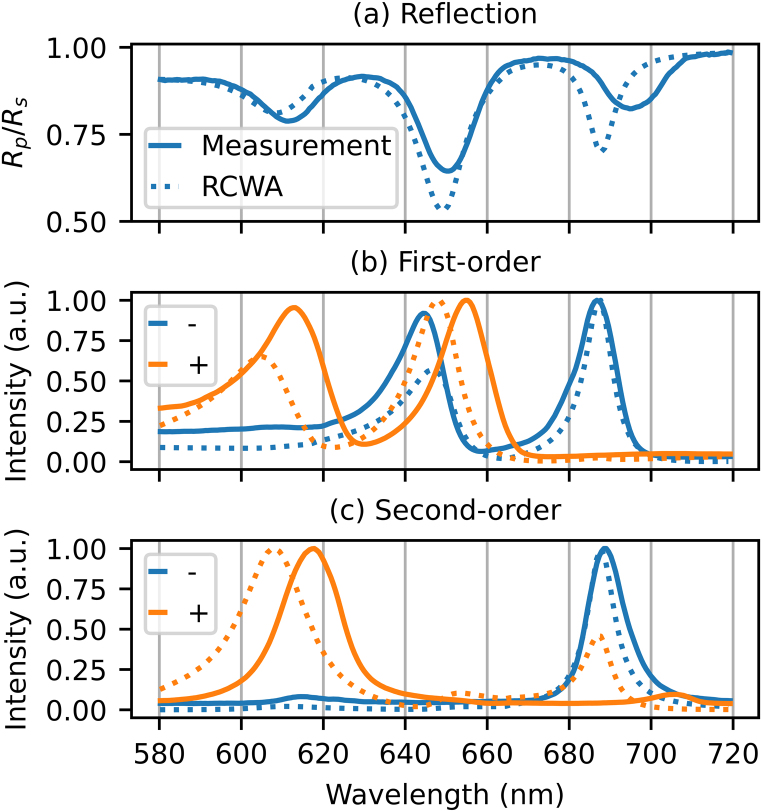
Measured and calculated spectra of the specular reflection, plus and minus, first- and second-order diffraction. (a) Measured (solid) and calculated (dashed) ratio of the reflection spectra of p and s-polarised incident light. (b and c) Measured (solid) and calculated (dashed) individually normalised spectra of plus (red) and minus (blue), first-(b), and second-order (c) diffraction, for p-polarised incident light. (For interpretation of the references to colour in this figure legend, the reader is referred to the web version of this article.)

White light spectroscopy of the segmented grating is performed, where the spectra of the zeroth- (reflection), first-, and second-order diffracted beams were recorded, for both p- and s-polarised light. Both the plus and minus orders of the non-zero diffraction orders are recorded. In addition, these spectra were simulated using Rigorous Coupled Wave Analysis (RCWA) numerical calculations [7]. The RCWA numerical calculations were performed with at least five hundred harmonics, to ensure convergence. Fig. 2 shows the measured and calculated spectra for these orders. The reflected spectrum is shown as the ratio of the p- and s-polarised reflected spectra, while the diffraction spectra shown are only for p-polarised light and are normalised to the maximum of each individual spectrum. The results of the white light spectroscopy for the plus and minus second-order diffraction are shown for completeness only. The measured strain-wave-induced changes in the second-order probe diffraction were also recorded but are not discussed in this paper.

### 3.1 Reflection

Fig. 2(a) shows the ratio of the measured (solid line) p- and s-polarised reflection spectra. The dashed line shows the ratio of the p- and s-polarised reflection spectra as calculated by the RCWA code. We note that the SPPs are only excited with p-polarised light. By plotting the ratio of reflection spectra for p- and s-polarised light, features common to both polarisations are suppressed, while SPPs are highlighted.

In Fig. 2(a), three dips, at 611, 650, and 695 nm, are visible in the measured spectra. Each one corresponds to an SPP resonance, which is explained in more detail in Appendix B. We note again that, in contrast to these results, a *non*-segmented grating typically only shows *one* SPP resonance, in lowest order [8], [9]. In the calculated spectrum, three dips are also present, but at the slightly different wavelengths of 608, 650, and 688 nm. Furthermore, the shapes and depths of the dips are somewhat different. The calculated dip at 608 nm, for example, is less deep than the measured resonance at 611 nm. At 650 nm, the calculated dip is much deeper than measured, and the dip at 688 nm is both narrower and deeper than the measured one. The difference between the calculation and the measurement may be due to deviations in the shape of the fabricated grating from the perfect rectangular shape used in the calculations.

### 3.2 First-order diffraction

The plus (red) and minus (blue) first-order diffraction spectra for p-polarised incident light are shown in Fig. 2(b). The solid lines are the measured spectra and the dashed lines are calculated with the RCWA code. The measured, plus first-order diffraction spectrum has peaks at 613 and 654 nm, while the calculated spectrum has peaks at 605 and 648 nm. The measured, minus first-order spectrum peaks at 645 nm and 687 nm, the calculated spectrum has peaks at 647 and 688 nm. Again, differences between calculated and measured spectra are attributed to deviations from a perfect rectangular shape of the grating used in the experiment.

### 3.3 Second-order diffraction

Fig. 2(c) shows the plus (red) and minus (blue) second-order diffraction spectra for p-polarised incident light. The solid lines represent the measured spectra and the dashed lines the calculated spectra. In contrast to the first-order diffraction spectra, only one peak is visible in the second-order spectra. This peak is at 617 nm for the measured, and at 608 nm for the calculated plus second-order diffraction spectrum. The RCWA calculation shows a second peak in the plus second-order at 687 nm, which is not present in the measured spectrum. The minus first-order has its peak at 689 nm for the measured spectrum and at 688 nm for the calculated spectrum. As before, the difference between calculated and the measured spectra are attributed to deviations in the shape of the fabricated grating from a perfect rectangle. It is known that such deviations have a stronger influence on higher diffraction orders [10], [11].

## 4 Surface plasmon polaritons

Surface plasmon polaritons (SPPs) of a specific (optical) frequency can be excited by light incident with a certain angle on a metallic grating, depending on the dielectric function of the metal and, more importantly, the grating period. The segmented grating is essentially a modulation of a short-period ‘plasmonic’ grating by a longer-period ‘alignment’ grating. As a result, ‘sideband’ gratings exist at the difference and the sum of the plasmonic and alignment (angular) spatial frequencies, which are $k_{p1}$ and $k_{A}$ respectively. Visible light can excite SPPs on the original plasmonic grating and the difference (with angular spatial frequency $k_{p2}$) and sum (with $k_{p3}$) gratings at the same incident angle of 27° with respect to the surface normal, but for different wavelengths. Table 1 shows the angular spatial frequency, associated grating period, the SPP resonance wavelength calculated via Eq. (B.2), and the measured SPP resonance wavelength, for each of these three gratings. For a more elaborate description of SPPs on the segmented grating, we would like to refer to Appendix B.

**Table 1:** Measured and calculated SPP resonance wavelengths of the ‘original’ plasmonic grating (with angular spatial frequency $k_{p1}$) and the two ‘sideband’ gratings ($k_{p2}$ and $k_{p3}$). The resonance wavelengths were measured at an incident angle of about 27° and were calculated using Eq. (B.2) for an incident angle of 27.7°.

Grating angular	Period	SPP resonance wavelength (nm )	
spatial frequency	(nm )	Calculation	Measurement
$k_{p1}=14k_{A}$	430	650	650(1)
$k_{p2}=13k_{A}$	463	695	695(1)
$k_{p3}=15k_{A}$	401	612	611(1)

### 4.1 SPP coupling to propagating light

Surface plasmon polaritons, when propagating along a surface, can couple to free-space light modes when a grating is present at the surface. In this subsection, we investigate in which directions light can couple out when SPPs are propagating along the surface of the segmented grating. This analysis is similar to that shown in [12].

**Fig. 3:**
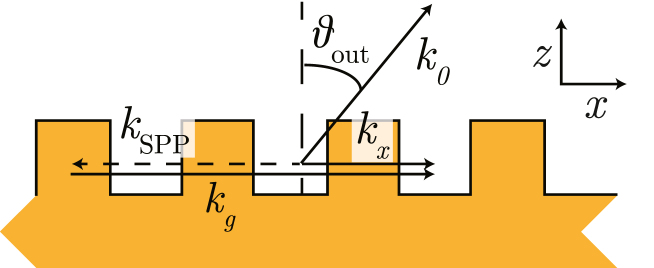
Sketch of the coupling of SPPs to free space light modes, which propagate in a direction with an angle $\theta_{\text{out}}$ with the z-axis and with wavevector $k_{0}$ using a sub-wavelength grating with angular spatial frequency $k_{g}$.

**Fig. B.17:**
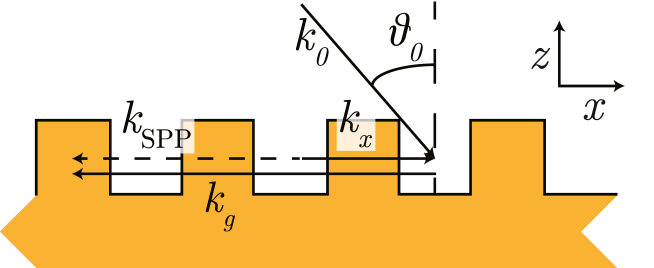
Schematic drawing of the excitation of SPPs by light, incident at an angle $\theta_{0}$ with the z-axis, and with wavevector $k_{0}$ using a sub-wavelength grating with angular spatial frequency $k_{g}$. $k_{x}$ is the projection of ${\overset{¯}{k}}_{0}$ onto the surface.

Consider a situation where an SPP is propagating along an Au/Air interface with a grating present, as shown in Fig. 3. In this situation, the SPP travels to the left, similar to Fig. B.17, and couples out to light propagating in free space in the x- and positive z-direction, at an angle $\theta_{\text{out}}$ with the z-axis, when the following condition is met:

(1) $$k_{\text{SPP}}=k_{0}\sin\theta_{\text{out}}-k_{g}.$$

This equation is very similar to Eq. (B.2), except that here, we describe the situation where light is coupled *out*.

As an example, let us consider SPPs excited on the grating with angular spatial frequency $k_{g}=k_{p1}$, incident at an angle $\theta_{\text{in}}$, and propagating along the segmented grating in the negative x-direction. In principle, three gratings with angular spatial frequency $k_{p1}$, $k_{p2}$, or $k_{p3}$ can be used to couple to a freely propagating mode. Using Eq. (1), the direction of the propagating light can be calculated. If we choose $k_{g}=k_{p1}$, and by substituting $k_{\text{SPP}}$ in Eq. (1) with the expression in Eq. (B.2), the angle $\theta_{out}$ can be calculated with,

(2) $$k_{1}\sin\theta_{0}-k_{p1}=k_{1}\sin\theta_{\text{out}}-k_{p1}.$$

It follows that $\theta_{\text{out}}=\theta_{\text{in}}$. The light propagates in the direction of the specular reflection.

If light couples out using a different grating, for example, on the grating with $k_{g}=k_{p2}=13k_{A}$, this results in:

(3) $$k_{1}\left(\sin\theta_{0}-\sin\theta_{\text{out}}\right)=k_{p1}-k_{p2}=14k_{A}-13k_{A}=k_{A}.$$

This equation can be recognised as the equation for diffraction from a phase grating with angular spatial frequency $k_{A}$. Thus, light coupled in on grating $k_{p1}$, is coupled out through the grating with angular spatial frequency $k_{p2}$. It will propagate in the same direction as the plus first-order diffraction *from the long period grating*. Similarly, light coupled out on the grating with $k_{g}=k_{p3}$ will have the same $k_{x}$ as the minus first-order diffraction from the long period grating. SPPs excited on the sideband gratings with $k_{p2}$ and $k_{p3}$ will also couple out through the three gratings, in directions which coincide with the diffraction orders from the long-period alignment grating. Table 2 summarises which SPPs at the three resonance wavelengths are allowed to ‘re-scatter’ in the direction of the lower order diffracted beams, from the 6020nm period alignment grating. Every SPP resonance can, in principle, couple to propagating modes using the three gratings which make up the segmented grating in lowest order.

The re-scattered light can also interfere with light directly diffracted from the long period grating. The EM-field experiences a phase shift when it couples to SPPs and also when SPPs couple to propagating waves [13], [14]. The exact phase shift is, unfortunately, not a-priori known. The interference is (implicitly) taken into account by the RCWA calculations, as it numerically solves the Maxwell Equations. Separating the contributions from the diffracted and the re-scattered light is, however, not possible.

**Table 2:** SPP coupling to propagating free-space propagating modes, calculated using the grating angular spatial frequencies shown in Table 1 and calculated using Eq. (1). The propagating modes are in the same direction as the diffraction orders, diffracted off the 6020 nm period grating. The table shows the diffraction order and the (vacuum) wavelengths of the SPPs which are allowed to re-scatter in the direction of that order.

Diffraction order	SPP resonance		
from 6020 nm period grating	wavelengths (nm)		
−2			695
−1		650	695
0	612	650	695
+1	612	650	
+2	612		

Looking at the results from the white light spectroscopy in Fig. 2, our simple analytical model, which takes only terms of the product of the Fourier series in lowest order into account, correctly predicts the number of peaks in each spectrum. The central wavelengths of the calculated peaks are within 8 nm of the measured peaks. More importantly, this analysis helps to understand how changes in the SPP resonances, induced by strain waves, can affect the diffraction from the long period grating.

## 5 Results

### 5.1 Reflection measurements

**Fig. 4:**
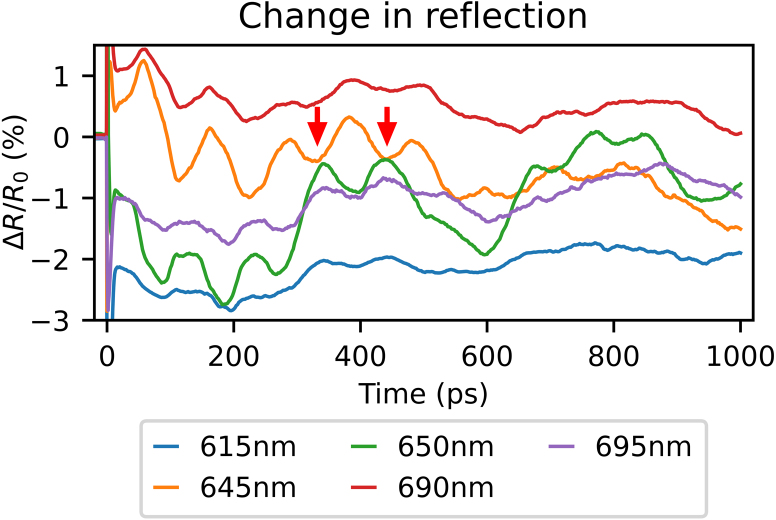
Measured probe-pulse reflection changes as function of time delay between the pump and probe pulse after excitation by the 400 nm central wavelength pump pulse, for selected probe wavelengths. Indicated with red arrows is the change in sign when measuring with a wavelength just below an SPP resonance, at 645 nm (orange curve), compared to measuring at the SPP resonance, at 650 nm (green curve). Note that sign changes can be seen for other time delays as well. (For interpretation of the references to colour in this figure legend, the reader is referred to the web version of this article.)

Fig. 4 shows the measured time-dependent pump-induced reflection changes, $\Delta R/R_{0}$, where $R_{0}$ is the unperturbed reflection, for selected wavelengths between 600 and 700 nm. For all wavelengths, a sharp peak or dip is seen for a time delay around 0 ps. For longer delays, an oscillatory signal is observed, consisting of short (<150ps) and long (∼400ps) period oscillations. The oscillation amplitude strongly depends on the probe wavelength. Furthermore, the short period oscillations change sign when probing with a wavelength at or just above the resonance wavelength, compared to a probe wavelength just below the resonance wavelength. This behaviour can most clearly be seen when comparing the trace measured using a probe wavelength of 650 nm (the SPP resonance wavelength) with the one measured at 645 nm. Red arrows in Fig. 4 indicate where this change of sign is most clearly seen. In addition to the oscillations, there is also an ‘offset’ present in the measurements for delays >0ps. This offset appears to decay to ∼0% reflection change on a time scale of several nanoseconds.

The sharp peak or dip observed around 0 ps delay time is caused by the effect that the fast heating and cooling of the electron gas has on the SPP resonance, similar to what was observed in [15], [16]. The electron gas cools by heating the lattice. This leads to a rapid expansion of the lattice, which generates several types of strain waves [17]. The strain waves manifest themselves as a time-varying oscillatory change in the optical reflection [18], [19]. The slowly varying offset is likely caused by the effect that the heated (and slowly cooling) material has on the SPP resonance via the thermo-optic effect [4]. We cannot completely exclude the possibility that the slowly varying offset could *partially* be caused by the effect of a surface acoustic wave at a acoustic frequency well below 1 GHz, associated with the periodicity of the alignment grating of 6020 nm. Only part of this surface-acoustic-wave period can be measured as the maximum time delay in our setup is limited to about 1 ns. The possible contribution from this surface acoustic wave cannot be separated from the thermally-induced change in the SPP resonance.

The oscillatory signal shows several periodicities and we are able to identify two of them [4]: Surface Acoustic Waves (SAWs, at about 2.6 GHz), and Longitudinal Waves (LWs, at 9.5GHz). SAWs are strain waves travelling along the surface, in the direction perpendicular to the grating lines. For our Au layer thickness, the LWs manifest themselves as an expansion and contraction of the whole Au layer and have a frequency determined by the speed of sound in Au and the layer thickness. In Section 6, we explain the physical nature of these different waves in more detail and how the LWs affect the probe reflection and diffraction.

**Fig. 5:**
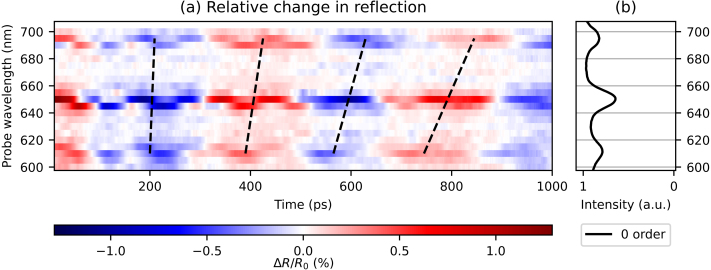
(a) 2D plot of the measured probe pulse reflection changes as function of time delay between the pump and probe pulse, where the slowly decaying background has been removed, for probe wavelengths between 600 and 700 nm. Indicated with black dashed lines is the increase in SAW period for increasing probe wavelength. (b) Ratio of measured p- and s-polarised reflection spectra. (For interpretation of the references to colour in this figure legend, the reader is referred to the web version of this article.)

To facilitate a comparison between the different traces, we have first removed the slowly-decaying background from each curve. This is done by subtracting a fitted decaying exponential function from these curves for times ≥10ps. The results are shown as a 2D plot in Fig. 5(a) where we plot the relative change in reflection $\Delta R/R_{0}$, indicated by the different colours, as a function of time (horizontal axis) after optical excitation, and as a function of probe wavelength (vertical axis). Blue indicates a decrease and red an increase of reflection with respect to the (removed) background. For ease of comparison, the ratio of the measured p- and s-polarised reflection spectra is shown on the right, in Fig. 5(b). The figure clearly shows that the reflection changes are strongest at probe wavelengths of 610, 650, and 695 nm, which correspond to the SPP resonances. Surprisingly, Fig. 5(a) clearly shows that the period of the SAW increases with increasing probe wavelength, as indicated by the black dashed lines.

**Fig. 6:**
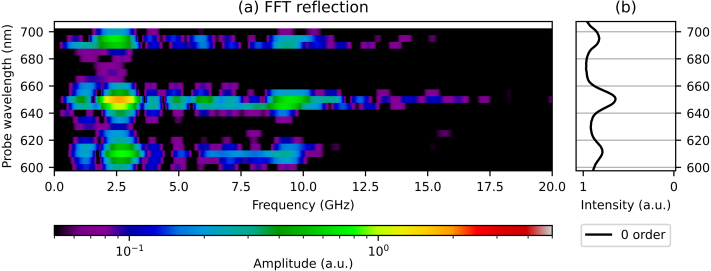
(a) 2D plot of the FFT spectra of the measured probe pulse reflection changes, for probe wavelengths between 600 and 700 nm. (b) Ratio of measured p- and s-polarised reflection spectra. (For interpretation of the references to colour in this figure legend, the reader is referred to the web version of this article.)

Several oscillatory signals, with different amplitudes, frequencies, and phases, are present in the data. To extract spectra from the strain-wave signals from the time-domain data, we calculate the FFT of the curves, with the slowly-varying background removed, zero-padded from about 1 to about 32 ns. In Fig. 6(a), we plot the amplitudes of the spectra, where the x-axis represents the strain-wave frequency, the y-axis the probe wavelength, and where the colour represent the amplitude of each frequency component (on a logarithmic scale). In Fig. 6(b), the ratio of the p- and s-polarised reflection spectra is plotted again for comparison. For most probe wavelengths, there is a peak around a strain-wave frequency of 2.6 GHz, which is attributed to SAWs. The SAW frequency slightly decreases for increasing probe wavelength, which can be seen more clearly in the time-domain data in Fig. 5 as an increasing oscillation period with increasing probe wavelength. For probe wavelengths close to the SPP resonances, an additional peak is observed around 9.5 GHz, corresponding to the LWs. By comparing the FFT amplitudes of the SAW and LW of the measurement with the smallest reflection changes, at the probe wavelength of 675 nm, to those of the measurement with the largest changes, at 650 nm, we find that the SAW amplitudes are increased by a factor of about 23 and the LW amplitudes by a factor of about 36.

In previous experiments on non-segmented gratings [4], a third type of strain wave was identified, in addition to the SAW and LW. This was the (Quasi-)Normal Mode (NM), a vibration of the grating lines, with a frequency between that of the SAWs and LWs. The NM was identified by its frequency [20], and we expect a NM frequency of about 8 GHz for the small-period grating line size of this segmented grating. This is a higher frequency than observed in previous experiments, as the (short-period) grating line dimensions are smaller than in the earlier experiments [4]. While the amplitudes of the spectra for strain-wave frequencies between the SAWs and LWs are not zero, at least for probe wavelengths close to the SPP resonances, there is no clear peak around 8 GHz. Thus, these oscillatory signals cannot be attributed to NMs, and their origin is currently not known.

### 5.2 Minus first-order diffraction measurements

**Fig. 7:**
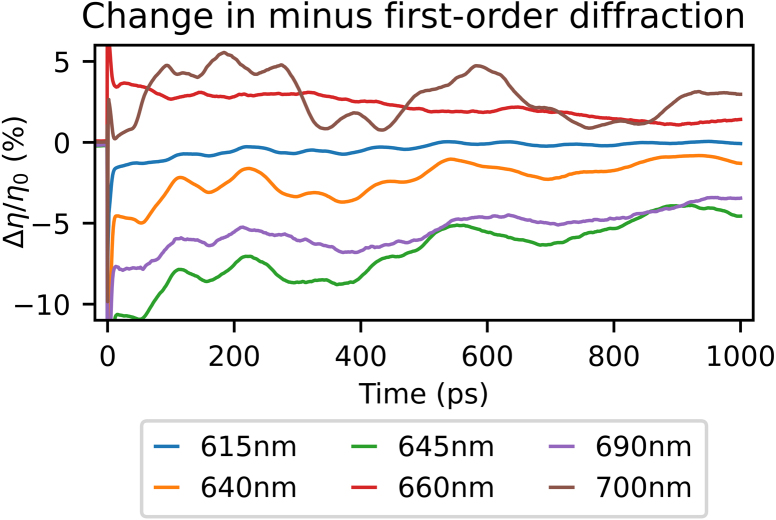
Measured changes in minus first-order probe *diffraction* as function of time delay between the pump and probe pulse after excitation by the 400 nm pump pulse, for selected probe wavelengths. (For interpretation of the references to colour in this figure legend, the reader is referred to the web version of this article.)

In our measurements, we observe first-, and second-order probe diffraction off the long-period alignment grating. The pump-induced relative changes in the minus first-order diffraction efficiency of the probe, $\Delta \eta /\eta_{0}$, where $\eta_{0}$ is the unperturbed diffraction efficiency, as a function of pump–probe delay time, are shown in Fig. 7, for selected probe wavelengths. Similar to the time-dependent reflection measurements, a sharp peak or dip is seen for all probe wavelengths around 0 ps time delay, and oscillatory signals with short and long periods are observed for longer delay times. A slowly-decaying thermal background is also present. In comparison to the reflection measurements, the strain-wave-induced changes in diffraction, as well as the diffracted signal from the initial thermal background are larger. In the reflection measurements, the maximum peak-to-peak change is about 2 %, while in the minus first-order diffraction the maximum peak-to-peak change is about 5 %. For probe wavelengths close to the SPP resonance wavelengths, a change in sign is again observed for the LW oscillations, similar to what is seen in the reflection measurements.

**Fig. 8:**
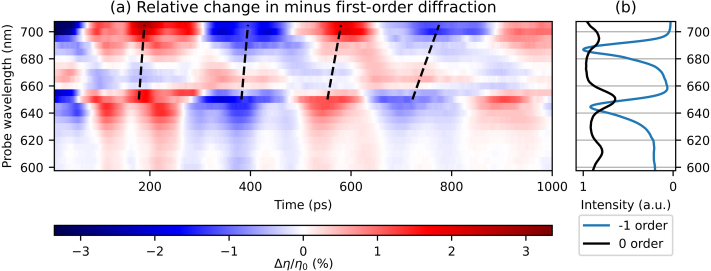
(a) 2D plot of the measured changes in minus first-order *diffraction* of probe light as function of time delay between pump and probe pulse, where the slowly decaying background is removed, for probe wavelengths between 600 and 705 nm. Indicated with black dashed lines is the increase in SAW period for increasing probe wavelength. (b) The ratio of measured p- and s-polarised reflection spectra and the minus first-order diffraction spectrum. (For interpretation of the references to colour in this figure legend, the reader is referred to the web version of this article.)

Fig. 8(a) shows the changes in diffraction as a function of pump–probe delay time for all probe wavelengths, where the decaying thermal background was removed in a similar manner as in the reflection measurements. Fig. 8(b) shows two measured spectra: the ratio of the p- and s-polarised reflection spectra and the minus first-order diffraction spectrum, all measured for an incident angle of 27° with respect to the surface normal. As in the reflection measurements, the long period SAW is clearly visible and has the largest amplitude. Interestingly, in contrast to the reflection measurements, strong signals are only seen around the two longer SPP resonances wavelength, at 650 and 695 nm. At the shortest SPP resonance wavelength of 610 nm, there is neither a peak in the diffraction spectrum (Fig. 8(a)), nor are there enhanced photo-acoustic signals. Furthermore, the peak wavelengths are slightly redshifted with respect to the peaks in the minus first-order diffraction spectrum, which are at 645 and 687 nm, as can be seen in Fig. 8(b). Fig. 8(a) also shows that the SAW period increases, similar as in the reflection measurements, when the probe wavelength increases from 650 to 705 nm. Interestingly, here the SAW also changes sign in between 660 and 675 nm, which was not observed in either the reflection measurement or in previous experiments on non-segmented gratings [4]. For probe wavelengths shorter than 645 nm, the shape of the traces remains similar, but the amplitude decreases with shorter probe wavelengths.

**Fig. 9:**
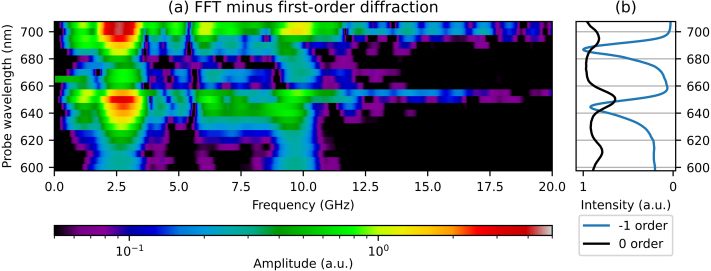
(a) 2D plot of the FFT of the measured changes in minus first-order diffraction of probe light, for probe wavelengths between 600 and 705 nm. (b) The ratio of measured p- and s-polarised reflection spectra and the minus first-order diffraction spectrum. (For interpretation of the references to colour in this figure legend, the reader is referred to the web version of this article.)

Fig. 9(a) shows the amplitude of the FFT of the strain-wave signals, as a function of strain-wave frequency and probe wavelength. Fig. 9(b) shows the reflection spectrum and the minus first-order diffraction spectrum. At all probe wavelengths, the peaks due to the SAWs and the LWs are clearly observed at 2.6 and 9.5 GHz, respectively. For probe wavelengths around the two longer wavelength SPP resonances, between 640 and 655 nm, and between 690 and 695 nm, there are also strain waves observed with a frequency between 6.0 and 7.5 GHz. As discussed in 5.1, these frequencies have not yet been identified. We note that the measured frequency associated with the LWs, hints at a small shift from about 9.9 GHz to about 9.2 GHz, when the probe wavelength is increased from 665 nm to 680 nm. Finally, we compared the FFT amplitudes of the SAW and LW with the largest changes in the minus first-order diffraction, at a probe wavelength of 700 nm, with the FFT amplitudes of the measurement with the largest *reflection* changes, measured at 650 nm. We find that the SAW induced diffraction change is enhanced by an additional factor of about 2.4 and by a factor of about 2 for the LW. We note this is an enhancement in the relative change in diffraction, but that the absolute intensity of the diffracted light is at least one order of magnitude smaller than that of the reflection.

### 5.3 Plus first-order diffraction measurements

**Fig. 10:**
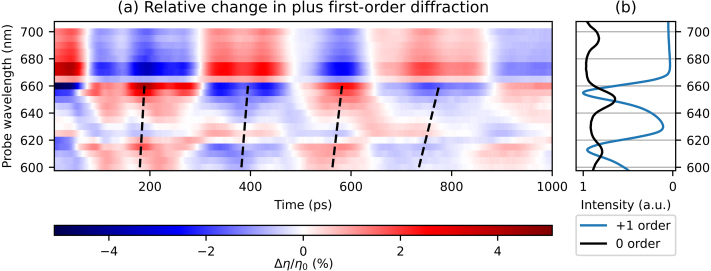
(a) 2D plot of the measured changes in *plus* first-order diffraction of probe light as function of time delay between pump and probe pulse, where the slowly decaying background is removed, for probe wavelengths between 600 and 700 nm. Indicated with black dashed lines is the increase in SAW frequency for increasing probe wavelength. (b) The ratio of measured p- and s-polarised reflection spectra and the plus first-order diffraction spectrum. (For interpretation of the references to colour in this figure legend, the reader is referred to the web version of this article.)

**Fig. 11:**
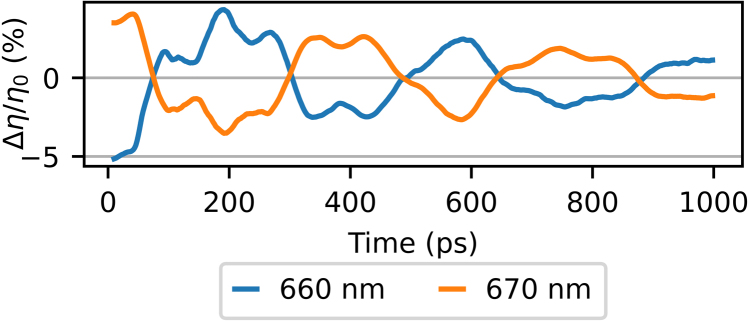
Comparison of the measured changes in plus first-order diffraction of probe wavelengths 660 and 670 nm, where the slowly-decaying background is removed, as function of pump–probe delay time. (For interpretation of the references to colour in this figure legend, the reader is referred to the web version of this article.)

The strain-wave-induced changes in the *plus* first-order diffraction are very similar to those in the minus first-order diffraction. Therefore, Fig. 10(a) directly shows the measured plus first-order diffraction changes as function of delay time for probe wavelengths between 600 and 700 nm, where the background has been removed in a similar manner as in previous 2D plots. Fig. 10(b) shows the ratio of the p- and s-polarised reflection spectra and the plus first-order diffraction spectrum. When looking at how the diffraction changes between different probe wavelengths, we observe a similar trend as in the minus first-order diffraction measurements. For example, in the plus first-order diffraction measurements, a sign change of the SAW is observed, between the two shorter wavelength SPP resonances, at 620 nm and at 640 nm. In the minus first-order measurements, two sign changes are also observed, the first at 660 nm and the second at 680 nm, again in between two SPP resonance wavelengths. We also note that the minus first-order diffraction spectrum has peaks at the two longer SPP resonance wavelengths, at 645 and 687 nm, whereas the *plus* first-order spectrum has peaks around the two shorter SPP resonance wavelengths, at 613 and 654 nm. Furthermore, the measurements with the probe wavelengths of 660 and 670 nm are completely opposite in sign, as is more clearly shown in Fig. 11.

**Fig. 12:**
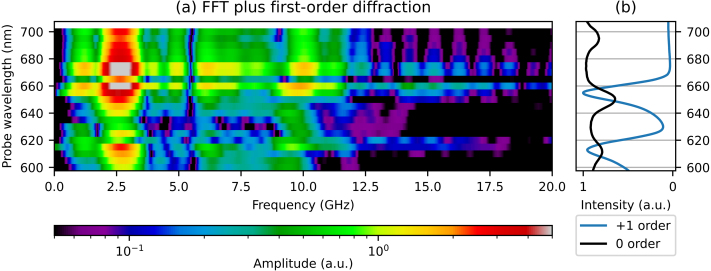
(a) 2D plot of the FFT of the measured changes in plus first-order diffraction of probe light, for probe wavelengths between 600 and 700 nm. (b) The ratio of measured p- and s-polarised reflection spectra and the plus first-order diffraction spectrum. (For interpretation of the references to colour in this figure legend, the reader is referred to the web version of this article.)

The FFT of the plus first-order time-dependent diffraction signals is shown in Fig. 12(a). Fig. 12(b) shows the reflection spectrum and the spectrum of the plus first-order diffracted beam. In Fig. 12(a), at all probe wavelengths, signals at the two frequencies corresponding to the SAWs and the LWs are observed. Again, there are also still unidentified contributions to the signal at frequencies between 6 and 7.5 GHz. Similar to the minus first-order diffraction measurements, we see a hint of a shift in the frequency of the LW signal, when probing with a wavelength between the SPP resonance wavelengths, between 625 and 645 nm. Here, the frequency shifts from about 9.9 to 9.4 GHz. The enhancement, when we compare the FFT amplitudes of the measurement with the largest plus first-order diffraction changes, at 670 nm, with the FFT amplitudes of the largest *reflection* changes (at a probe wavelength of 650 nm), is a factor of about 3.3 for the SAW and a factor of about 2.6 for the LW. Again, we note that these are measured as relative changes and that the intensity of the diffracted light is at least one order of magnitude smaller than the intensity of the reflection.

## 6 Discussion

In the experiments, we see two different dominant strain-wave frequencies, at about 2.6 and 9.5 GHz. As in our previous experiments, where we probed around a SPP resonance with a non-segmented Au grating of similar Au layer thickness [4], we attribute the two frequencies to Surface Acoustic Waves (SAWs), excited on the short-period grating(s), and Longitudinal Waves (LWs), respectively. In the first part of this section, we discuss the generation and physical properties of the different waves. In the second part, we discuss how these strain waves can influence the probe signal.

### 6.1 Strain waves

#### 6.1.1 Surface acoustic waves

Surface acoustic waves travel along a surface, and can be optically excited if there are spatially periodic inhomogeneities in either the excitation profile [21], [22], [23] or in the surface topography [17]. In similar, earlier experiments, we have concluded that the excited SAWs are likely to be shear waves [4]. This may have been wrong. The observed SAWs may in fact be Rayleigh waves. These propagate along the free surface of a solid, with a phase velocity typically somewhat lower than the transverse speed of sound [24]. Furthermore, they have both a longitudinal and a transversal motion component and their amplitude typically decays exponentially with depth, at least for a single thick layer of material. The precise nature of these waves on our segmented grating containing multiple layers is currently not known.

In all experiments, we observe a SAW frequency of around 2.6 GHz. The time-domain data show that the SAW period is shorter for shorter probe wavelengths, and longer for longer probe wavelengths. This is indicated by black dashed lines in Figs. 5a, 8a, and 10a. This is also observed in the second-order diffraction measurements (not shown). This behaviour was not seen in our earlier experiments on non-segmented gratings [4]. We note that the excitation conditions for the excitation of strain waves with the 400 nm pump pulse are identical for all measurements across different reflection and diffraction orders. However, rigorous numerical simulations are necessary to further investigate the different SAW frequencies. These simulations should include the excitation by the 400 nm pump pulse using near-field calculations, 2D election-energy diffusion, the time-dependent strain-wave formation and propagation on these square-wave gratings, and the diffraction of the probe pulse. This is currently beyond the scope of this paper in which we want to focus on LWs that may be used to detect buried gratings.

#### 6.1.2 Longitudinal waves

Longitudinal waves are strain waves travelling between the free surface of the grating and the Au/SiO_2_ interface. Due to the relatively large penetration depth of energy in the Au layer [2], [25], the 172(3) nm thick Au layer heats up relatively homogeneously, even though the *optical* penetration depth is only a fraction of the layer thickness. The homogeneous heating leads to the generation of two strain waves, one at the Air/Au interface and another at the Au/SiO_2_ interface, travelling in opposite direction. These two counter-propagating strain waves result in a standing wave, causing the layer to expand and contract. The frequency of the cycle of expansion and contraction is determined by dividing the speed of sound in Au by twice the thickness of the layer, $v_{\text{Au}}/2d_{\text{layer}}$, as both strain waves need to travel back to the same interface where they are generated. A measurement (shown in Appendix C) on an unstructured part of the sample, where only LWs are excited, shows a frequency of 9.3(2) GHz. This matches with the peak at a frequency of about 9.5 GHz, supporting the identification of these waves as LWs.

### 6.2 Strain-wave-induced reflection and diffraction changes

Most types of strain waves change the density of the material as they propagate. In this case, strain waves change the material density of the Au and, as the electrons respond quasi-instantaneously [26], also the electron density, $N_{e}$. The change in electron density will shift the plasma frequency, given by $\omega_{p}={\left(e^{2}N_{e}/\epsilon_{0}\;m\right)}^{1/2}$, where e is the electron charge, $\epsilon_{0}$ is the vacuum permittivity, and m the effective electron mass. The shifting plasma frequency changes the relative permittivity of Au, and, according to Eq. (B.1), the wavevector and the resonance wavelength of the SPP as well. Thus, strain waves can shift the central wavelength of an SPP resonance [26], [27], [28], [29]. They may also change the grating shape, such as its amplitude or grating line width.

As explained in [4], a strain wave affecting the central wavelength of an SPP resonance can be recognised by a change in sign (or a π-phase shift) in the measured oscillation, when comparing a trace measured using a probe wavelength just below an SPP resonance with one equal to or just longer than the SPP resonance wavelength. This behaviour is also present in the segmented grating experiment for the frequencies associated with LWs, and is indicated by red arrows in Fig. 4 and Fig. 7, for the measured probe reflection and minus first-order diffraction changes, respectively. However, in the 2D plots, this is not clearly visible, as the LW oscillations are superimposed on the SAW oscillations, which are stronger. By spectrally filtering the obtained FFT spectra of the reflection and first-order diffraction changes to remove the SAW and transforming the filtered spectra back to the time domain, the sign changes of these oscillations become more clearly visible. We have used a bandpass filter with a cut-on frequency of 8.5 GHz, just below the frequencies associated with the LWs at 9.5 GHz, and a cut-off frequency of 75 GHz, to suppress noise. Furthermore, the applied bandpass filter has sharp, but smooth transitions near the cut-on and cut-off frequencies, to reduce filtering artefacts.

**Fig. 13:**
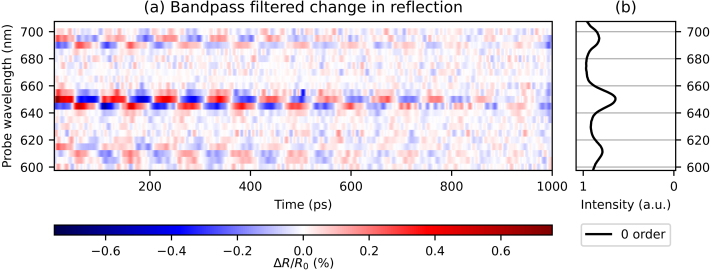
(a) 2D plot of the bandpass filtered changes in reflection, as function of time delay between pump and probe pulse, for probe wavelengths between 600 and 700 nm. Cut-on frequency: 8.5 GHz, cut-off frequency: 75 GHz (b) The ratio of measured p- and s-polarised reflection spectra. (For interpretation of the references to colour in this figure legend, the reader is referred to the web version of this article.)

**Fig. 14:**
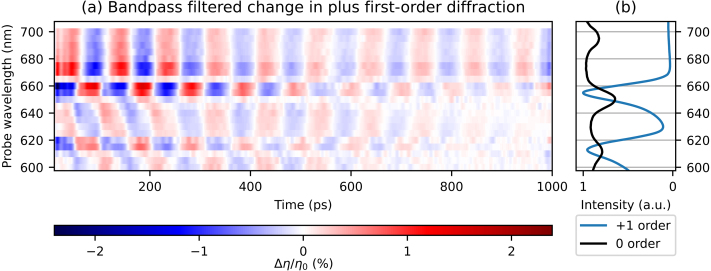
(a) 2D plot of the bandpass filtered changes in plus first-order diffraction, as function of time delay between pump and probe pulse, for probe wavelengths between 600 and 700 nm. Cut-on frequency: 8.5 GHz, cut-off frequency: 75 GHz (b) The ratio of measured p- and s-polarised reflection spectra and the plus first-order diffraction spectrum. (For interpretation of the references to colour in this figure legend, the reader is referred to the web version of this article.)

Figs. 13(a) and 14(a) show the bandpass filtered, time-dependent signals for all probe wavelengths, for the reflection and the plus first-order diffraction, respectively. Again, Fig. 13(b) shows the reflection spectrum and Fig. 14(b) shows both the reflection and plus first-order diffraction spectra. Starting with the filtered reflection changes (Fig. 13(a)), we observe two clear sign changes, at probe wavelengths of 650 nm and 695 nm, and a third, much fainter one at 615 nm. These probe wavelengths are equal to, or just longer than, the SPP resonance wavelengths of 611, 650, and 695 nm. In Fig. 14, where the filtered plus first-order signals are shown, four sign changes are observed, at the probe wavelengths of 610, 625, 650, and 665 nm. Of these, 610 and 650 nm are very close to SPP resonances, but the other two are not. Furthermore, the *absence* of sign changes for the bandpass filtered signals in the probe wavelength range of 680 to 700 nm is also surprising. In Section 4, we have explained that the peaks in the diffraction spectra can be explained by the multiple angular spatial frequencies present in the segmented grating, if we assume that SPPs excited through one grating can couple out through another. In our analysis, SPPs do not couple out in the direction of the plus first-order diffraction spectrum for wavelengths between 680 and 700 nm. Thus, at wavelengths where we observe a sign change associated with an SPP resonance in the *reflection* measurements, we do not always observe this change in sign in the plus first-order diffraction at the same probe wavelengths. A similar behaviour is observed in the bandpass filtered minus first-order signals (not shown) in the wavelength range of 610 to 630 nm, where the reflection measurements show a sign change.

When comparing the starting phases of identical probe wavelengths of the bandpass filtered reflection and plus first-order diffraction signals, we observe another interesting feature. For example, the signal at the probe wavelength of 645 nm starts with a relative decrease in reflection, but shows an increase in the plus first-order diffraction. At 650 nm, where the sign is changed in both reflection and plus first-order diffraction, the reflection increases and the plus first-order decreases. This inverse behaviour of reflection and diffraction signals at the same wavelength is also observed near the probe wavelengths of 615 nm in the plus first-order diffraction and near the sign changes at 650 nm and 695 nm of the minus first-order (not shown). Thus, for LWs, we see that an decrease in reflection correlates with an *increase* in diffraction for probe wavelengths near SPP resonances, and we conclude that the reflection decreases and the diffraction increases as more light is coupled to SPPs, for probe wavelengths close to SPP resonances. We note that the *relative* changes in diffraction can be greater than the relative changes in reflection, but that the *absolute* changes in reflection are larger, as the unperturbed reflection is much larger than the unperturbed diffraction efficiency. For example, at the probe wavelength of 655 nm, the measured, unperturbed reflection at this wavelength is about 70 % and the measured, unperturbed diffraction efficiency, $\eta_{0}$, is about 3 %. Furthermore, the diffraction efficiency changes drastically as the probe wavelength changes, meaning that the large relative changes in diffraction, at probe wavelengths 670 nm and 675 nm are not as large as those around 655 nm, in absolute terms.

**Fig. 15:**
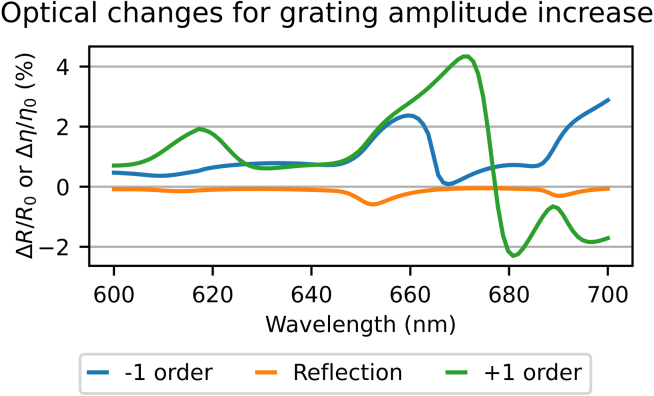
Calculated relative reflection and diffraction changes for a 100 pm increase in grating amplitude, as a function of probe wavelength. (For interpretation of the references to colour in this figure legend, the reader is referred to the web version of this article.)

Strain waves can also affect the reflection and diffraction due to changes of the grating shape. If the grating shape change is known, the resulting changes in reflection and diffraction can also be calculated with the RCWA code. As an example, Fig. 15 shows the calculated, relative change in reflection and first-order diffraction as a function of wavelength, assuming a 100 pm increase in grating amplitude. The increase in grating amplitude yields a decrease in reflection, for all wavelengths. Furthermore, the decrease is largest at about 613, 652, and 690 nm. These are just a bit longer than the SPP resonance wavelengths calculated with the RCWA code at 608, 650, and 688 nm. For the minus first-order diffraction, the diffraction increases for all wavelengths calculated, and the relative increase peaks at 660 and 700 nm, where the calculated, absolute diffraction efficiency is very low (see Fig. 2(b)). Similar peaks are observed around 617 and 670 nm in the relative change in plus first-order diffraction efficiency. Again, the calculated, absolute diffraction efficiency is very low for these wavelengths. Surprisingly, the change in plus first-order diffraction quickly becomes negative for wavelengths longer than 677 nm.

It is unlikely that actual strain waves will uniformly change the grating amplitude, as we have used in the calculations, but the calculations illustrate that a grating deformation can result in opposite behaviour at different probe wavelengths within the same (non-zero) diffraction order. In other words, the effect of a grating shape change, in this example a height increase, does not give a diffraction increase for all wavelengths. For a more detailed understanding, finite element methods could potentially be used to calculate (time-dependent) grating deformations, which could be used as input for RCWA calculations to determine the changes in reflection and diffraction, perhaps even including the change in permittivity induced by strain.

## 7 Conclusion

We have studied the strain-wave-induced reflection and diffraction changes of a segmented grating, as a function of pump–probe delay time. The segmented grating consists of a short-period grating modulated by a longer-period grating, leading to two sidebands in the spatial frequency domain around the angular spatial frequency of the short-period grating. Surface plasmon polaritons (SPPs) can be excited on the small-period grating and on the two ‘sideband’ gratings, leading to three separate SPP resonances. The presence of three gratings leads to strongly enhanced diffraction off the longer-period grating, for wavelengths close to the SPP resonance wavelengths. This is caused by SPPs re-scattering light in the same direction as light directly diffracted off the longer-period grating.

The time-dependent, strain-wave-induced reflection and diffraction changes were studied using probe light with wavelengths covering the three SPP resonances on the segmented grating. In reflection, the strongest strain-wave-induced oscillatory signals were observed at the SPP resonance wavelengths. Surface acoustic waves (SAWs) and longitudinal waves (LWs) were identified by their frequency. In reflection, the enhancement when probing near SPP resonance wavelengths was about a factor of 23 for SAWs and 36 for LWs, respectively. In the first-order diffraction measurements, oscillatory signals similar to the reflection measurements were observed, but the relative changes of the diffraction signals were larger by a factor of 3.3 for SAWs and by a factor of 2.6 for LWs, compared to the reflection measurements.

## Declaration of Competing Interest

The authors declare that they have no known competing financial interests or personal relationships that could have appeared to influence the work reported in this paper.

## Figures and Tables

**Fig. A.16:**
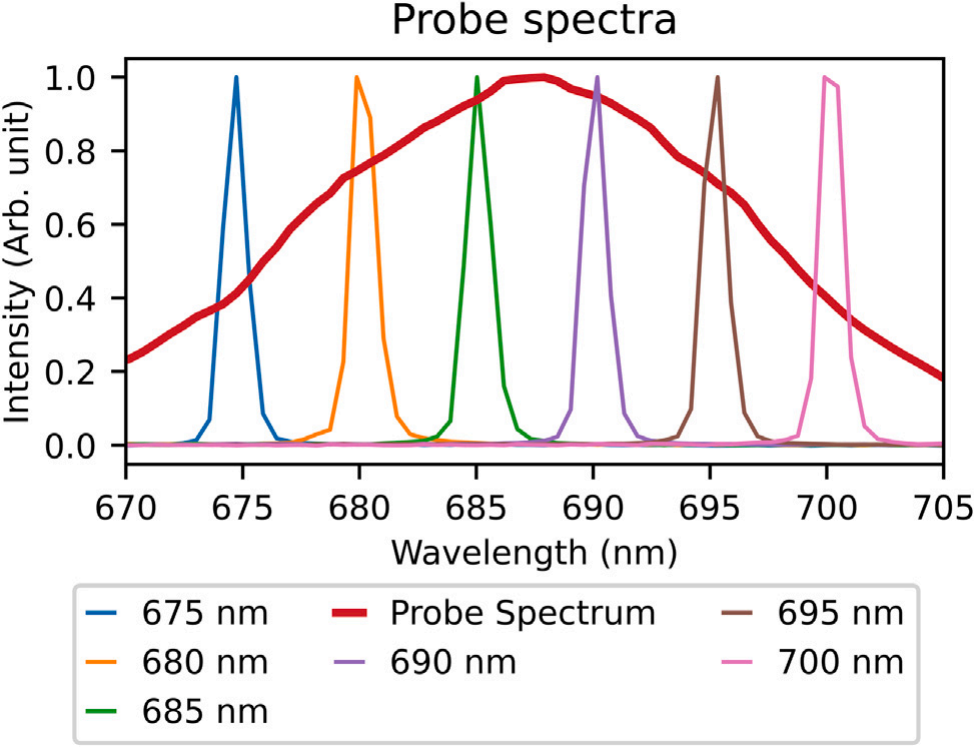
Example of the unfiltered probe spectrum (red line), with a 687 nm central wavelength, together with six probe spectra, obtained by filtering the original probe spectrum. All curves have been normalised to their maximum. (For interpretation of the references to colour in this figure legend, the reader is referred to the web version of this article.)

**Fig. C.18:**
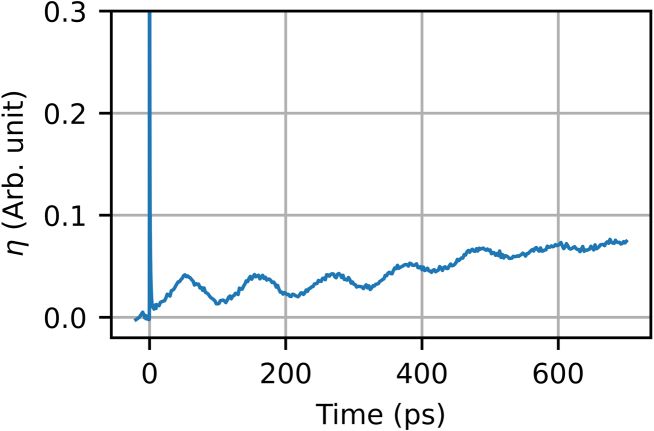
Measured time-dependent diffracted signal from an unstructured part of the sample, after excitation by two synchronised 400 nm pulses, creating a transient grating. The measurement diffraction is normalised to the electron peak at 0 ps delay.

## Data Availability

Data will be made available on request.

## Appendix A Experimental setup

A schematic view of the experimental setup is shown in Fig. 1(a). The output of an amplified Ti:Sapphire laser system (Astrella, Coherent) with a repetition rate of 1 kHz, a pulse length of about 35 fs, a central wavelength of 800 nm and a pulse energy of 6 mJ is split into two beams using a 85:15 (R:T) beam splitter.

The transmitted, weaker part of the beam is frequency doubled to 400 nm using a β-barium borate (BBO) crystal. It is reflected by several dichroic mirrors to filter out residual 800 nm light and passes a computer-controlled variable optical delay line. A mechanical chopper, synchronised with the 1 kHz pulse train, blocks every other pulse, reducing the repetition rate to 500 Hz. A lens is used to weakly focus the light onto the sample, such that the spot diameter is about 500 µm. The pump light is p-polarised, perpendicular to the grating lines, and incident at an angle of about 7° with respect to the surface normal. The pump light does not excite SPPs.

The reflected, stronger part of the output of the laser system is used as input for a three-stage Optical Parametric Amplifier (OPA, TOPAS, Light Conversion). We tune the output of the OPA in a range from 1200 to 1410 nm. Another BBO crystal is used to frequency-double this to a range of 600 to 705 nm. Using a lens (f=20cm), the probe beam is weakly focused onto the sample, to an elliptic spot with axes of about 140µm and 80µm. The probe light is also p-polarised and incident at an angle of approximately 27° with respect to the surface normal. For this incident angle, the probe pulse excites SPPs on the sample at wavelengths of approximately 611, 650, and 695 nm. The fluence of the pump pulse is 8.5 mJ cm^−2^, about 60 % of the energy is absorbed by the sample.

The probe beam diffracts off the 6020 nm period grating and several diffraction orders are present. An aperture selects one order, which is collimated by a lens and is used to illuminate a blazed grating (GR25-1205, Thorlabs). The diffracted light is focused onto a movable slit. Using the slit, only a 1.5–2.0 nm wide band of wavelengths is transmitted to the photodiode detector (PDA100A2, Thorlabs), out of the full 15 nm FWHM bandwidth of the frequency-doubled pulses from the OPA. As an example, Fig. A.16 shows the unfiltered spectrum of a 687 nm central wavelength pulse from the frequency-doubled OPA output, together with six filtered probe spectra, all normalised to their individual maximum. The (specular) reflected, and the plus and minus first-order diffracted beams were measured as a function of time delay between the pump and probe pulses. Within the broad spectrum of the probe pulse, of which the central wavelength is varied through adjustment of the OPA, five to six wavelength intervals of 2 nm are sequentially measured. The energy of the probe pulse was adjusted when the central wavelength of the output of the OPA was changed, but was kept constant when measuring within the bandwidth of the frequency-doubled output of the OPA.

White light spectroscopy is performed using a fibre-coupled broadband white light source (AVALIGHT-HAL-S-MINI, Avantes) and a fibre-coupled spectrometer (AvaSpec-ULS-RS-TEC, Avantes). The light, coming from the white light source, is collimated (in free space), and passes through a wire-grid polariser. It is focussed onto the sample, at an angle of about 27°, similar to the incident angle of the probe pulse in the photo-acoustic experiments. The polariser allows us to investigate the difference between p- and s-polarised incident light, as only p-polarised light can excite SPPs. Using an aperture, a single diffraction order is selected, that is collimated and coupled into the fibre going to the spectrometer.

## Appendix B Light coupling to surface plasmon polaritons

In Au, the angular wave number of SPPs, $k_{\text{SPP}}=\left|{\overset{¯}{k}}_{\text{SPP}}\right|$, for optical frequencies corresponding to a wavelength of 600 nm or longer, can be approximated by [30]:

(B.1) $$k_{\text{SPP}}\approx \frac{\omega }{c}\sqrt{\frac{\epsilon_{\text{Au}}^{\prime }\epsilon_{\text{env}}}{\epsilon_{\text{Au}}^{\prime }+\epsilon_{\text{env}}}},$$

where ω is the optical angular frequency, c the speed of light, $\epsilon_{\text{Au}}^{\prime }$ is the real part of the permittivity of Au at ω, and $\epsilon_{\text{env}}$ is the permittivity of the environment, which is $\epsilon_{\text{env}}\approx 1$ for air.

Fig. B.17 shows a drawing of how SPPs are excited using a grating. Propagating light, incident at angle $\theta_{\text{in}}$ with respect to the surface normal, can be coupled to SPPs if the following condition is met [30]:

(B.2) $$k_{\text{SPP}}=k_{0}\sin\theta_{\text{in}}+mk_{g},$$

where $k_{0}\sin\theta_{\text{in}}=k_{x}$ is the projection of the wavevector of the incident light, ${\overset{¯}{k}}_{0}$, onto the sample surface, $k_{0}=\left|{\overset{¯}{k}}_{0}\right|=2\pi /\lambda$ is the wavenumber, $k_{g}=2\pi /\Lambda_{g}$ is the angular spatial frequency of the grating with period $\Lambda_{g}$, and m is an integer. For our sub-wavelength period plasmonic gratings and used wavelengths, usually m=−1.

The segmented grating, however, consists of a plasmonic grating ($\Lambda_{p1}=2\pi /k_{p1}=\text{430}\;\text{nm}$) modulated by the alignment grating ($\Lambda_{A}=2\pi /k_{A}=\text{6020}\;\text{nm}=14\Lambda_{p1}$). The height of such a segmented grating as a function of the position x, assuming a 50 % duty cycle and a perfect square grating, can written be as:

(B.3) $$f_{\text{sg}}\left(x\right)=\frac{h}{4}\left(1+f_{p1}\left(x\right)\right)\left(1+f_{A}\left(x\right)\right),$$

where (B.4)

$$f_{p1}\left(x\right)=\frac{4}{\pi }\underset{n=1,3,5,\ldots }{\sum }\frac{\sin\left(nk_{p1}x\right)}{n}==\left\{\begin{array}{cc} +1\; & \text{for}l\Lambda_{p1}<x<\left(l+\frac{1}{2}\right)\Lambda_{p1},\\ 0\; & \text{for}x=\frac{1}{2}l\Lambda_{p1},\\ -1\; & \text{for}\left(l+\frac{1}{2}\right)\Lambda_{p1}<x<\left(l+1\right)\Lambda_{p1}, \end{array}\right)$$

wherel=0,±1,±2,…in all cases,

and (B.5)

$$f_{A}\left(x\right)=\frac{4}{\pi }\underset{n=1,3,5,\ldots }{\sum }\frac{\sin\left(nk_{A}x\right)}{n}==\left\{\begin{array}{cc} +1\; & \text{for}l\Lambda_{A}<x<\left(l+\frac{1}{2}\right)\Lambda_{A},\\ 0\; & \text{for}x=\frac{1}{2}l\Lambda_{A},\\ -1\; & \text{for}\left(l+\frac{1}{2}\right)\Lambda_{A}<x<\left(l+1\right)\Lambda_{A}, \end{array}\right)$$

wherel=0,±1,±2,…in all cases,

are the Fourier series describing the square gratings with angular spatial frequencies $k_{p1}$ and $k_{A}$, respectively. By using only the lowest order terms of both series, Eq. (B.3) can be approximated by: (B.6)

$$f_{\text{sg}}\left(x\right)\approx \frac{h}{4}\left[1+\frac{4}{\pi }\sin\left(k_{p1}x\right)\right]\left[1+\frac{4}{\pi }\sin\left(k_{A}x\right)\right]==\frac{h}{4}[1+\frac{4}{\pi }\sin\left(k_{p1}x\right)+\frac{4}{\pi }\sin\left(k_{A}x\right)+\;\frac{16}{\pi^{2}}\sin\left(k_{p1}x\right)\sin\left(k_{A}x\right)].$$

Here, the first two sines after the last equal sign represent the original gratings. The cross-term is the result of the modulation (multiplication) of the plasmonic grating by the alignment grating. This term can be rewritten as: (B.7)

$$\sin\left(k_{p1}x\right)\sin\left(k_{A}x\right)=\frac{1}{2}\{\cos\left[\left(k_{A}-k_{p1}\right)x\right]-\;\cos\left[\left(k_{p1}+k_{A}\right)x\right]\}.$$

The modulation thus leads to two additional angular spatial frequencies, $k_{p2}=\left|k_{A}-k_{p1}\right|$ and $k_{p3}=\left|k_{p1}+k_{A}\right|$. This is mathematically similar to the generation of sidebands in radio-frequency amplitude modulation. Furthermore, as $k_{p1}=14k_{A}$ (i.e. the plasmonic angular spatial frequency is the 14th harmonic of the alignment angular spatial frequency), the sidebands are at $k_{p2}=13k_{A}$ and $k_{p3}=15k_{A}$, which can also been seen as the 13th and 15th harmonic of the alignment angular spatial frequency.

Light can couple in on all gratings present at the Au surface, as long as Eq. (B.2) holds. For the segmented grating, this means that light can couple to SPPs using the three gratings with angular spatial frequencies $k_{p1}$, $k_{p2}$, and $k_{p3}$.

Using Eq. (B.2) and using the permittivity for Au, calculated from the n- and k-values obtained via ellipsometry, light with a wavelength of 650 nm couples to SPPs on the grating with angular spatial frequency $k_{p1}$ (which is the *original*, non-segmented plasmonic grating) for an incident angle of 27.7°, with respect to the surface normal. Using this incident angle, the wavelengths required to couple light to SPPs on the two other plasmonic ‘sideband’ gratings can be calculated. For the same incident angle, this leads to SPP resonance wavelengths at 612 nm (for $k_{p2}$) and 695 nm (for $k_{p3}$). Table 1 lists the grating spatial frequencies expressed in terms of the long period alignment grating $k_{A}$, the corresponding spatial period, and the calculated and measured SPP resonance wavelength.

The calculated SPP resonance wavelengths are close to the measured ones. The incident angle used in the calculation (27.7°) is slightly different from the incident angle in the measurement (27°). This difference may be due to the fact that, strictly speaking, Eq. (B.2) is only valid for flat surfaces and is only a good approximation for grating amplitudes smaller than about 7 nm [31].

## Appendix C Transient grating measurement

Since we had some doubts about the accuracy with which the thickness of the Au layer was initially determined, an additional measurement on the flat part of the sample, where no grating is present, was performed. Fig. C.18 shows the measured, normalised, time-dependent diffraction, η, of a 655 nm pulse, after excitation by *two* 400 nm pump pulses. These pulses create a transient grating at the sample, with a pitch of about 3 µm. The measurement shows an oscillatory signal with a period of 107(2) ps, corresponding to a frequency of 9.3(2) GHz. This corresponds to the LW frequency measured at the segmented grating. We are thus led to conclude that the thickness of the Au layer is 172(3) nm, assuming the speed of sound in the layer is still 3240 m s^−1^, rather than the 192 nm that was measured using a profiler (Tencor P-7, KLA).
